# An optimized thermodynamics integration protocol for identifying beneficial mutations in antibody design

**DOI:** 10.3389/fimmu.2023.1190416

**Published:** 2023-05-19

**Authors:** Zizhang Sheng, Jude S. Bimela, Maple Wang, Zhiteng Li, Yicheng Guo, David D. Ho

**Affiliations:** ^1^ Aaron Diamond AIDS Research Center, Columbia University Vagelos College of Physicians and Surgeons, New York, NY, United States; ^2^ Zuckerman Mind Brain Behavior Institute, Columbia University, New York, NY, United States

**Keywords:** thermodynamics integration, antibody design, antibody 10-40, SARS-CoV-2, molecular dynamics simulation

## Abstract

Accurate identification of beneficial mutations is central to antibody design. Many knowledge-based (KB) computational approaches have been developed to predict beneficial mutations, but their accuracy leaves room for improvement. Thermodynamic integration (TI) is an alchemical free energy algorithm that offers an alternative technique for identifying beneficial mutations, but its performance has not been evaluated. In this study, we developed an efficient TI protocol with high accuracy for predicting binding free energy changes of antibody mutations. The improved TI method outperforms KB methods at identifying both beneficial and deleterious mutations. We observed that KB methods have higher accuracies in predicting deleterious mutations than beneficial mutations. A pipeline using KB methods to efficiently exclude deleterious mutations and TI to accurately identify beneficial mutations was developed for high-throughput mutation scanning. The pipeline was applied to optimize the binding affinity of a broadly sarbecovirus neutralizing antibody 10-40 against the circulating severe acute respiratory syndrome coronavirus 2 (SARS-CoV-2) omicron variant. Three identified beneficial mutations show strong synergy and improve both binding affinity and neutralization potency of antibody 10-40. Molecular dynamics simulation revealed that the three mutations improve the binding affinity of antibody 10-40 through the stabilization of an altered binding mode with increased polar and hydrophobic interactions. Above all, this study presents an accurate and efficient TI-based approach for optimizing antibodies and other biomolecules.

## Introduction

Antibodies are immune system proteins that recognize versatile foreign- and self-biomolecules. Antibody biotherapeutics have been growing fast for the treatment and prophylaxes of infectious diseases, cancer, and autoimmune diseases (1–3). Compared to small molecule drugs, therapeutic monoclonal antibodies have multiple advantages such as high potency and specificity, metabolic stability, and low antigenicity (4). Many therapeutic antibodies require further improvement in specificity and binding affinity through mutagenesis of antibody residues at or close to the antigen binding site (5–7). Computational algorithms, promising for the cost-efficient identification of beneficial amino acid mutations, are now in high demand (8). Currently, many physics- and knowledge-based (KB) algorithms have been developed for structure-based antibody optimization (9, 10). Most computational approaches score the effects of mutations by computing the relative binding free energy (RBFE) difference between the wildtype and mutant states (7, 10–13). Despite many antibodies being successfully optimized by computational approaches (7, 14–16), the predicted RBFE has a weak correlation with experimental data (17), resulting in a low success rate of identifying affinity-enhancing mutations.

Thermodynamic integration (TI) is an attractive alchemical free energy (AFE) algorithm that predicts RBFE of small molecule ligands with high accuracy (Pearson’s r ~0.8 and root mean square error (RMSE) ~1kcal/mol) (18, 19). Whether TI can be applied to protein design has not been thoroughly investigated. TI uses molecular dynamics (MD) simulation and statistical mechanics to detect free energy alterations in biomolecule systems caused by mutations in a small subset of atoms (20). Briefly, TI uses an alchemical transformation to gradually mutate the selected residue to another amino acid through multiple steps in the presence and absence of a receptor, with a coupling parameter λ (ranging from zero to one) controlling the pathway of transformation. The free energy difference between states, ΔG^0^_TI_, is calculated by integrating the generalized force along the transformation pathway. TI then uses the thermodynamic cycle to compute the RBFE difference (ΔΔG) between the wildtype and mutant states. With the improvement in the force field and sampling algorithm, TI performs comparably to another AFE methodology, free energy perturbation (FEP) (18, 19), which has been sophisticatedly optimized in commercial software FEP+ (9, 21). But FEP+ is not open-source and cannot be parallelized on a large scale (due to tokens).

AFE algorithms have advantages over KB algorithms in calculating the RBFE of protein-protein interaction by more precisely describing many factors contributing to the interaction energy. For example, contributions of remote conformational modulation, protein flexibility, solvation, water-mediated interaction, cofactors, post-translational modifications, and ions are poorly approximated or not incorporated by KB algorithms. TI and other physics-based methodologies solve the above issues by monitoring energy changes of proteins and cofactors in an explicit solvent environment with molecular mechanistic force fields, capturing protein dynamics with high accuracy. Thus, TI has the potential for a robust and accurate prediction of the RBFE of protein mutations.

Severe acute respiratory syndrome coronavirus 2 (SARS-CoV-2), the virus causing the ongoing COVID-19 pandemic, continues to evolve new variants that evade immune recognition (22, 23). Neutralizing antibodies (nAbs) that can tolerate viral mutations are critical for therapeutics and vaccine efficacy (24, 25). 10-40, a nAb isolated from a COVID-19 convalescent donor, broadly neutralizes sarbecoviruses by recognizing a highly conserved epitope on the receptor binding domain (RBD) of the viral spike to block cellular entry (26). 10-40-like or class nAbs - originates from a similar V(D)J recombination and binds a similar epitope - represent one major component of the SARS-CoV-2 infection and vaccine elicited antibodies that can broadly neutralize sarbecoviruses (27). However, compared to nAbs targeting other RBD sites, all isolated 10-40-like antibodies show relatively weak potency against the SARS-CoV-2 omicron variants of concern (26, 28). Whether the neutralization potency of 10-40-like antibodies can be improved against the circulating omicron subvariants is unclear.

In this study, we developed a TI protocol with a substantially improved accuracy for predicting beneficial mutations, which is superior to KB methods. A pipeline incorporating both TI and KB methods was established to perform saturation mutagenesis. The pipeline successfully identified beneficial mutations that improve both the binding affinity and neutralization potency of antibody 10-40. Molecular dynamics (MD) simulation revealed the structural basis of the synergistic effects of three beneficial mutations. This study developed and validated a TI-based approach for antibody improvement.

## Results

### An optimized TI protocol for predicting the relative binding affinity of antibody mutations

**Figure 1:**
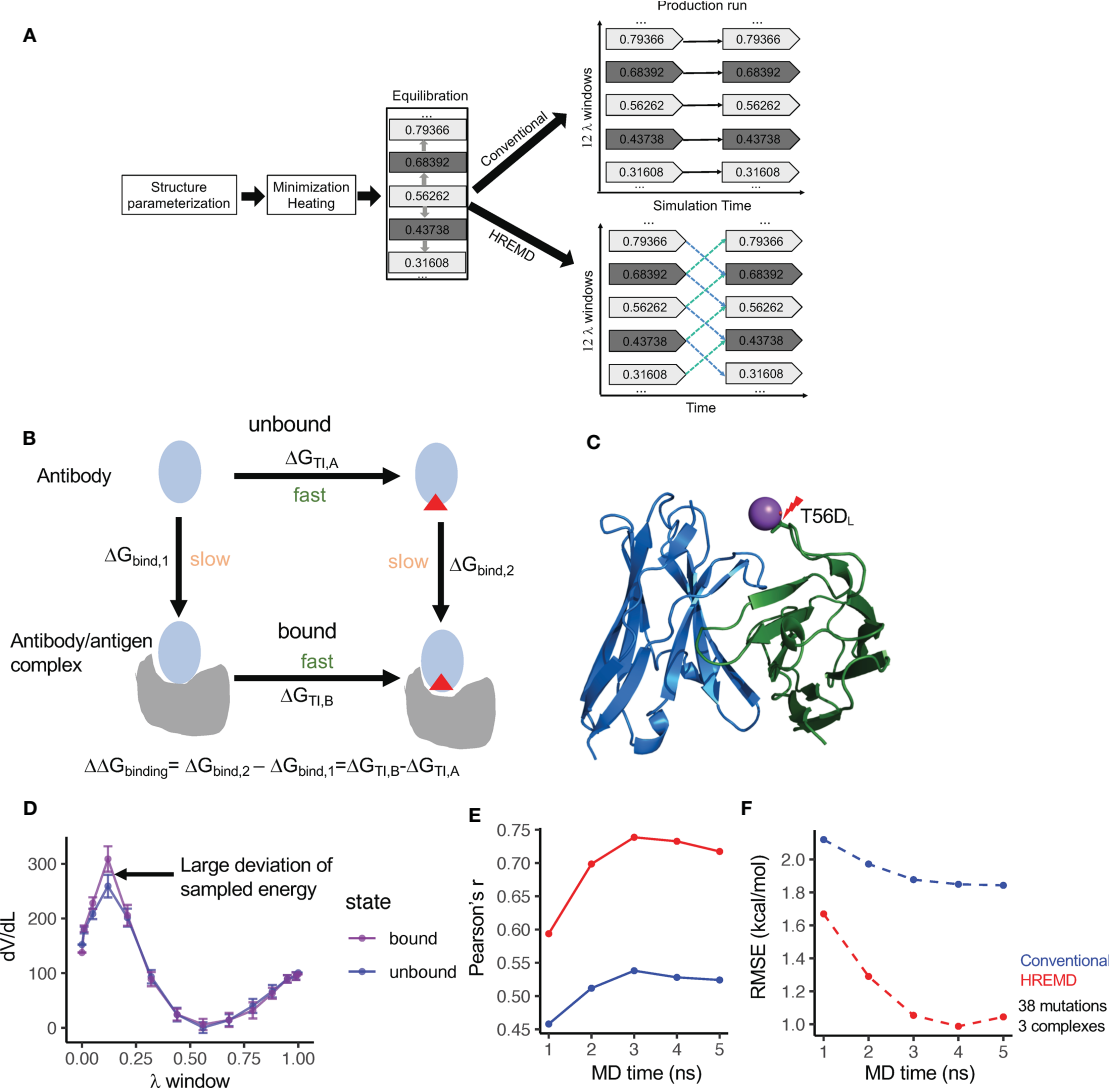
Diagram and the performance of thermodynamics integration protocols. **(A)** Diagram of the conventional and Hamilton-replica exchange MD TI protocols. **(B)** Diagram of the alchemical thermodynamics cycle for free energy calculation. **(C)** Clash between Na+ and S56D_L_ (PDBID: 2BDN) in MD with the conventional TI protocol leads to a large free energy spike (see **Figure S1A**). **(D)** HREMD TI protocol contains λ windows with large dV/dL deviation between antigen- bound and unbound MD runs. **(E)** Correlation of experimental measured binding free energy change (ΔΔG) and ΔΔG predicted by conventional and HREMD TI protocols with different MD simulation lengths. **(F)** Root mean square error (RMSE) between experimental measured ΔΔG and ΔΔG predicted by conventional and HREMD TI protocols with different MD simulation lengths.

To evaluate the performance of TI on predicting the RBFE of antibody mutations, we applied the conventional TI protocol (19) with 12 λ windows and the single topology approach, developed on small molecule ligand/receptor systems, to predict the RBFE of 38 mutations from three antibody/antigen systems (**Figures 1A, B**). Briefly, the TI protocol performs energy minimization, heating from 100K to 298K, system relaxation, equilibration, and production of MD (See details in Methods). The experimentally determined structures were used as the starting structures for mutagenesis and energy minimization (**Table S1**). The last snapshot of the previous step was used as the starting coordinate of the next step.

The 38 mutations include a balanced number of beneficial and detrimental mutations, all of which are within or peripheral to the epitope-paratope interface (**Table S1**). The analysis revealed that when mutating to negatively charged amino acids, the mutated residue forms clash with positively charged ions in the solution at one or a few λ windows, resulting in large jumps in the λ-derivative of the total energy (dV/dL) and the inaccurate prediction of the RBFE (**Figures 1C**, **S1A**). The incorporation of the recently developed smooth step function (18), which weighs dV/dL using a specified formula, substantially reduces the energy spike and improves the predicted RBFE (**Figure S1B**). Despite that, the predicted RBFEs of some mutations still deviated from the experimental RBFEs by more than 1.5kcal/mol. Further analysis showed that the predicted RBFEs with large errors tend to harbor λ windows with significant dV/dL deviation between the antibody-bound and -unbound systems (**Figure 1D**). Excluding such λ windows [identified using deviation from mean dV/dL by one standard deviation (SD)] further improved the prediction performance (**Figure S1C**). By incorporating the above strategies, the conventional TI protocol was applied to predict the RBFE of the 38 mutations with 5 nanosecond (ns) production run per λ window. The results showed that the performance of the conventional TI protocol converges in 3 to 4ns of MD simulation, which is comparable to the convergence time for predicting small molecule RBFE (19).

The conventional TI protocol showed a Pearson’s r of approximately 0.55 and a root mean square deviation (RMSE) of approximately 1.8kcal/mol (**Figures 1E, F**), which is not comparable to its performance on a small molecule RBFE prediction and is not ideal for antibody design. Because antibody/antigen complexes have system sizes much larger than small molecule/receptor complexes, we suspect that the conformation sampling of the antibody/antigen complexes may be inadequate using conventional MD. Thus, we applied the Hamilton replica exchange MD (HREMD) (29, 30), which allows for the exchange of intermediate states between adjacent λ windows during MD simulation to enhance sampling convergence. The results showed that HREMD substantially improved the performance of TI (Pearson’s r 0.74 and RMSE 1.05kcal/mol with 3ns MD) (**Figures 1E, F**).

We further used the one antibody-antigen system (human MCP-1/antibody 11K2, 2BDN, **Table S1**) to examine the impacts of MD length (up to 10ns), the number of λ windows, and the sizes of the waterbox on the accuracy of the HREMD TI protocol. The results showed that comparable ΔΔG RMSEs were obtained with 3ns and 10ns MD (**Figure S1D**), suggesting that a longer MD may not improve the accuracy. Increasing the number of λ windows from 12 to 16 reduced both Pearson’s r and RMSE. Changing the size of the waterbox from 6Å to 10Å increased Pearson’s r but reduced RMSE (**Figure S1D**). By considering both the computation time and accuracy, HREMD TI with 3ns MD, 12 λ windows, and 6Å waterbox were performed for the downstream studies.

The above performance was evaluated by averaging the RBFEs of each mutation from two independent TI runs. Our analysis showed that each single run can still reach high accuracy (3ns HREMD Pearson’s r 0.72 and 0.65) with a mean deviation of 0.90kcal/mol between runs. This suggests that a single TI run has a reasonable accuracy, which can be used for mutational screening and will save substantial computation time.

Above all, we found that our strategies significantly improved the performance of TI on antibody/antigen complexes and identified settings optimal for both the accuracy and efficiency of HREMD TI, which will be used for downstream studies.

### The performance of the HREMD TI protocol on a large dataset

To further examine the accuracy of the HREMD TI protocol, we established a curated dataset of 225 point mutations from 15 antibody/antigen complexes from both public databases (SKEMPI and AB-BIND: 171 mutations from 10 complexes) and literature (54 mutations from 5 complexes) (6, 10, 31, 32) (**Table S1**). These antibody/antigen complexes were chosen because each has mutations to diverse types of amino acids and has both favorable and deleterious mutations. In total, 55 favorable (ΔΔG< -0.5kcal/mol) and 119 deleterious mutations (ΔΔG>0.5kcal/mol) were included. The cutoff of 0.5kcal/mol was used to account for the uncertainty of experimental binding affinity measurements (9). A total of 36 mutations are from antigens and the rest are from antibodies. Mutations both within and away from the binding interfaces were included (**Table S1**).

**Figure 2:**
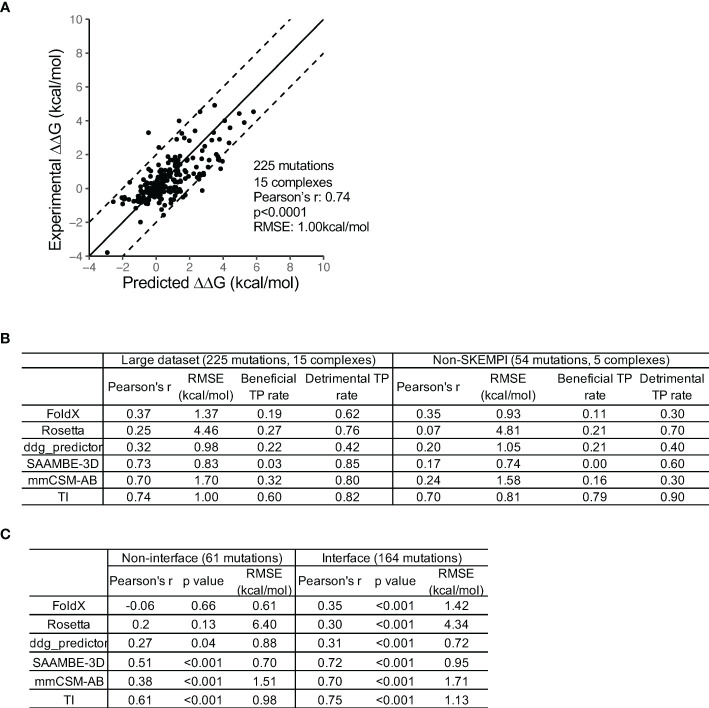
Comparison of the performance of TI and five knowledge-based computational methods. **(A)** A high prediction accuracy was obtained by the HREMD TI protocol. **(B)** TI outperforms the five KB algorithms at discriminating beneficial and detrimental mutations on the large and non-SKEMPI mutation datasets. The KB methods predict detrimental mutations with higher accuracy than beneficial mutations. The non-SKEMPI dataset does not include mutations in the SKEMPI database which is used to train knowledge-based algorithms. **(C)** TI outperforms the five KB algorithms at predicting RBFE of both antibody-antigen interface and non-interface mutations.

The result showed a single run HREMD TI to have a high accuracy (Pearson’s r 0.74 and RMSE 1.00kcal/mol) (**Figure 2A**). Among the 225 mutations, 51 mutations were predicted by HREMD TI to have ΔΔG absolute error greater than 1.3kcal/mol. Further dV/dL distribution analysis revealed 21 of the 51 mutations to have single or multiple λ windows significantly deviating between the antibody-bound and -unbound systems (identified using deviation from mean dV/dL by one SD). The exclusion of the deviated λ windows improved the RMSE of the 21 mutations from 5.35 to 2.81kcal/mol (**Figure S2C**), confirming the effectiveness of the strategy in improving prediction accuracy.

We then compared the performance of HREMD TI to five KB algorithms [FoldX (12), Rosetta (33), ddG-predictor (34), SAAMBE-3D (13), and mmCSM-AB (10)]. For the 225 mutation dataset, the comparison revealed that HREMD TI performed the best, followed by SAAMBE-3d and mmCSM-AB (**Figure 2B**). Because the mutations from SKEMPI and AB-BIND were used to train the machine learning algorithms ddG-predictor, SAAMBE-3D, and mmCSM-AB, we compared their performance using the 54 literature mutations collected from *in vitro* and *in vivo* antibody maturation studies. The result showed that the correlation coefficients of ddG-predictor, SAAMBE-3D, and mmCSM-AB substantially decreased to approximately 0.2. The performance of FoldX is comparable between the 225 and 54 datasets (**Figure 2B**) as well as a previous study with a larger testing dataset (17). To further compare the performance of TI to FEP+, we predicted the RBFE of 21 VRC01 mutations against the gp120 resurfaced stabilized core 3 (RSC3), which showed an accuracy comparable to FEP+ (**Figure S2E**) (9).

Because the goal was to identify beneficial mutations for antibody improvement, we further compared the accuracies of the KB and TI algorithms in predicting beneficial and deleterious mutations. The analysis showed that all algorithms predicted deleterious mutations with higher success rates than beneficial mutations (**Figure 2B**). HREMD TI achieved the best accuracy at predicting beneficial mutations while KB methods classified many beneficial mutations as neutral. HREMD TI also performed better at predicting the RBFEs of non-interface mutations than KB methods (**Figure 2C**), suggesting that TI can be used to explore a larger mutational space.

The high success rate of KB methods at predicting deleterious mutations suggests that KB methods can be used to exclude deleterious mutations in the mutation screening process. Thus, the high efficiency of the KB method and the high accuracy of the HREMD TI method can be integrated to identify beneficial mutations.

### Improving the binding affinity of antibody 10-40

**Figure 3:**
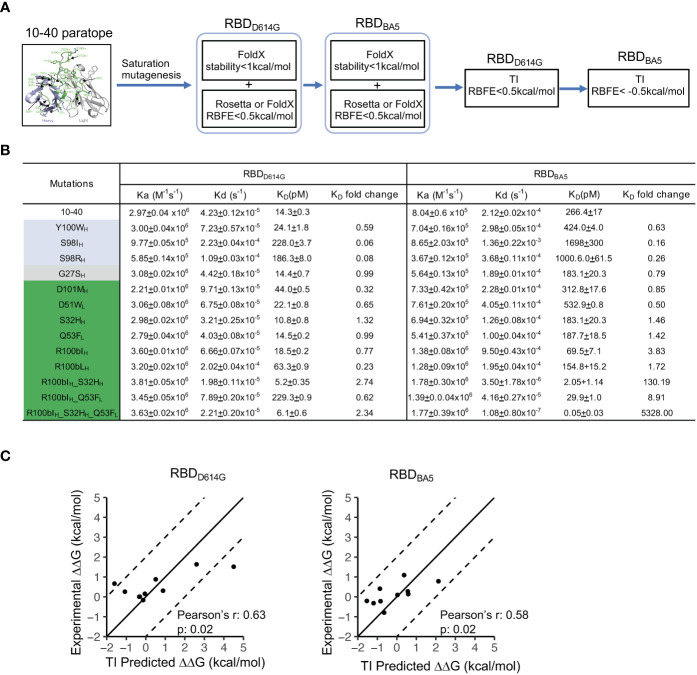
TI predicted beneficial mutations improve the binding affinity of antibody 10-40 against SARS-CoV-2 RBD_D614G_ and RBD_BA5_. **(A)** Antibody 10-40 beneficial mutation screening scheme. FoldX and Rosetta are used to exclude detrimental mutations against RBD_D614G_ and RBD_BA5_, and TI is used to identify beneficial mutations against RBD_BA5_. **(B)** Binding affinities of antibody 10-40 mutants against SARS-CoV-2 RBD_D614G_ and RBD_BA5_ measured by plasmon surface resonance (SPR). TI- predicted detrimental, neutral, and beneficial mutations are colored light blue, gray, and green, respectively. Data are shown with mean and standard deviation from triplicate. **(C)** Correlation of experimentally measured binding free energy change (ΔΔG) and ΔΔG predicted by HREMD TI protocol for 10-40 mutants against RBD_D614G_ (left) and RBD_BA5_ (right).

To validate our hypothesis that KB and TI methods can be combined to efficiently find beneficial mutations *via* saturation mutagenesis, we established a pipeline to improve the binding affinity of antibody 10-40 against the RBD from the circulating omicron subvariant BA5 (RBD_BA5_), without impairing the binding affinity against the wildtype RBD (RBD_D614G_) (**Figure 3A**). Because the 10-40/RBD_BA5_ complex structure is unavailable and a conformation change is observed at epitope positions of 370 to 376 in the antibody- free state (35), we modeled the 10-40/RBD_BA5_ complex and performed 20ns MD simulation. The complex structure from the last snapshot of the MD simulation was used for screening beneficial mutations against RBD_BA5_. Using the experimentally determined structure of 10-40/RBD_D614G_ and the MD-generated 10-40/RBD_BA5_, the pipeline first used FoldX and Rosetta to perform saturation mutagenesis at all 28 paratope positions to identify neutral and beneficial mutations to both RBD_D614G_ and RBD_BA5_ (see methods). TI was then applied to the obtained list of mutations to exclude deleterious mutations to RBD_D614G_ and to identify beneficial mutations to RBD_BA5_. Ten mutations, including four predicted to be deleterious or neutral by TI but to be beneficial by FoldX and/or Rosetta and six of the top predictions beneficial to RBD_D614G_ and/or RBD_BA5_ by TI, were synthesized and their binding affinities against RBD_D614G_ and RBD_BA5_ were measured (**Figure 3B**; **Table S3**).

The surface plasmon resonance (SPR) measurement showed that four of the six beneficial mutations (S32H_H_, R100bI_H_, R100bL_H_, and Q53F_L_) predicted by TI enhanced the binding affinity to RBD_BA5_ without substantially impairing the binding affinity to RBD_D614G_ (**Figure 3B**). The deleterious mutations predicted by TI were also validated by the SPR results, suggesting that TI can identify false positives predicted by FoldX and Rosetta (**Table S3**). Interestingly, TI predicted that adding S32H_H_ and Q53F_L_ to R100bI_H_ further improves the binding affinity of antibody 10-40 to RBD_BA5_ (**Table S3**). We then produced the combination mutants, and the SPR measurement revealed a strong synergy between the three mutations, with the triple mutations improving RBD_BA5_’s binding affinity by over 5,000-fold (**Figure 3B**). Overall, the TI predictions correlated with experimental data with Pearson’s correlation coefficients of approximately 0.6 (**Figure 3C**).

### The structural basis of the synergistic effects between S32H_H_, R100bI_H_, and Q53F_L_

**Figure 4:**
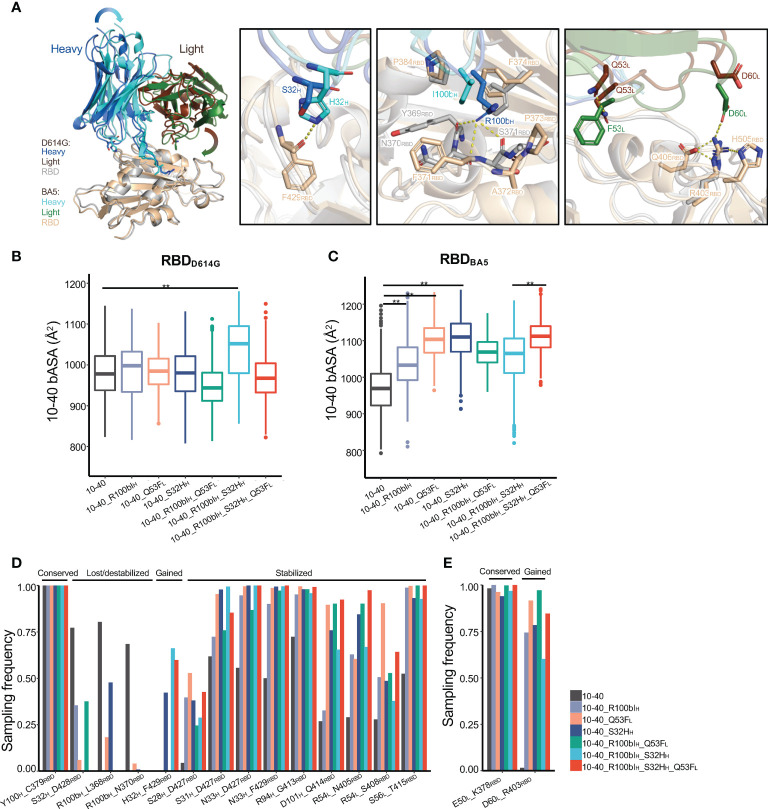
The structural basis of antibody 10-40 binding affinity improvement by S32H_H_, R100bI_H_, and Q53F_L_. **(A)** The MD simulation of 10-40 with triple mutations (S32H_H_, R100bI_H_, and Q53F_L_)/RBD_BA5_ shows substantial heavy and light chain conformation change compared to the 10-40/RBD_D614G_ complex. Each S32H_H_ (second panel), R100bI_H_(third panel), and Q53F_L_ (fourth panel) alter the local polar interactions between 10-40 and RBD. Hydrogen bonds and salt bridges are shown with dashed lines. **(B)** MD simulations of 10-40 mutants complexed with RBD_D614G_ show minor changes in the buried surface area between epitope and paratope except for the combination of S32H_H_ and R100bI_H_. **(C)** MD simulations of 10-40 mutants complexed with RBD_BA5_ show significant changes in the buried surface area of the paratope. **(D)** Multiple hydrogen bonds between the RBD_BA5_ epitope and paratope are stabilized by S32H_H_, R100bI_H_, and Q53F_L_. **(E)** 10-40 mutants form a new salt bridge with RBD_BA5_. Kolmogorov–Smirnov test is used to compare the significance of difference. P values less than 0.01 are labeled with **.

To understand the mechanism of synergy between S32H_H_, R100bI_H_, and Q53F_L,_ we performed MD simulations of antibody 10-40 with individual and combination mutation complexed with both RBD_BA5_ and RBD_D614G_. Overall, compared to the wildtype 10-40/RBD_D614G_ complex, the three mutations induced both local and global conformation changes to recognize RBD_BA5_ but not RBD_D614G_ (**Figures 4A**, **S2B**). Consequentially, for each mutation, we observed altered polar interactions in the 10-40/RBD_BA5_ complex (**Figure 4A** right).

The three mutations did not change the buried accessible surface area (bASA) between antibody 10-40 and RBD_D614G_ except the R100bI_H_ and S32H_H_ double mutant (**Figure 4B**), whose paratope bASA increased by approximately 100Å^2^, which is coincident with the 2.7-fold increase in binding affinity (**Figure 3B**). Surprisingly, for the 10-40/RBD_BA5_ complex, the three mutations induced similar conformational changes (**Figure S2B**), resulting in an increased paratope bASA (**Figure 4C**); the contribution was mostly by enhanced light chain interaction with the RBD_BA5_ (**Figure 4A**). Furthermore, individual and combinations of S32H_H_, R100bI_H_, and Q53F_L_ stabilized multiple hydrogen bonds and salt bridges between paratope and epitope in the 10-40/RBD_BA5_ complex but not the 10-40/RBD_D614G_ complex (**Figures 4D, E**, **S2C, D**). Above all, S32H_H_, R100bI_H_, and Q53F_L_ induced similar conformational changes for RBD_BA5_ recognition. The increased bASA and polar interactions are consistent with the improved binding affinity. The synergy between the three mutations is probably through additive stabilization of the altered binding mode.

### The neutralization potency of antibody 10-40 variants

**Figure 5:**
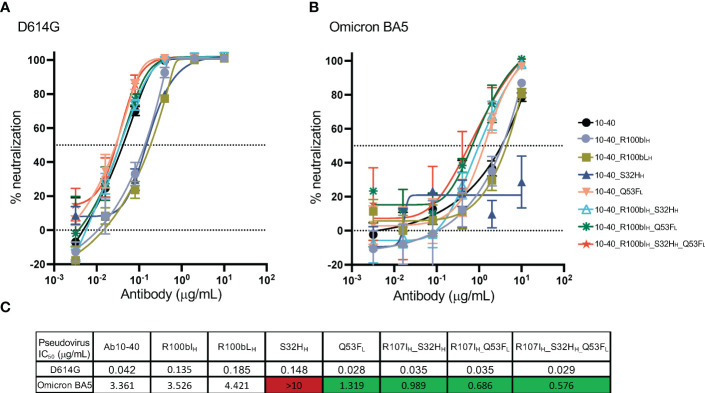
Pseudovirus neutralization potency of antibody 10-40 mutants. **(A)** The neutralization potency of antibody 10-40 mutants against the SARS-CoV-2 D614G strain. **(B)** The neutralization potency of antibody 10-40 mutants against the SARS-CoV-2 omicron BA5 subvariant. **(C)** The mean IC_50_ of antibody 10-40 mutants. The triple mutations improve the potency of 10-40 against both D614G and omicron BA5 subvariant. Antibody mutants with improved and reduced potency are highlighted in green and red, respectively. Data are shown with mean and standard deviation from three replicates.

A pseudovirus neutralization assay was performed to measure the potency of antibody 10-40 variants against the SARS-CoV-2 D614G and omicron BA5 strains (**Figures 5A, B**). For the D614G strain, each S32H_H_, R100bI_H_, and R100bL_H_ decreased the potency by approximately 3-fold (**Figure 5C**). Q53F_L_ and the combination of S32H_H_, R100bI_H_, and Q53F_L_ slightly improved the D614G neutralization potency, which is consistent with the binding affinity results. For the BA5 subvariant, R100bI_H_ and R100bL_H_ did not affect the potency. S32H_H_ reduced the potency substantially, which cannot be explained by the minor change in the binding affinity. Nonetheless, Q53F_L_ and the double and triple mutations increased the potency by approximately 3 to 5-fold.

## Discussion

In this study, we performed a comprehensive investigation on the performance of thermodynamic integration for predicting the relative binding affinity of antibody and antigen mutations and identified optimized parameter settings to substantially improve its accuracy and efficiency. The new TI protocol outperforms knowledge-based methods in discriminating beneficial and deleterious mutations. With the increased computing power of modern GPUs, TI provides an accurate and feasible approach for both the optimization of biomolecules and the prediction of the effects of protein substitutions in natural evolution. We found that the incorporation of HREMD and the statistical correction strategies to TI increase the accuracy of a single TI run, which substantially shortened the time to screen a large number of mutations. It takes about 24 GPU hours to calculate the RBFE of one mutation in the 10-40/RBD complex on an NVIDIA GTX 1080TI GPU, while the most accurate FEP+ protocol (100ns MD per λ window) takes approximately a week (9, 21). The run time of TI could be shortened to approximately 12 hours or less using the most advanced GPUs. Thus, the improved TI protocol alone or in combination with KB methods could be broadly applied to *in silico* mutational scanning.

This study reveals that the smooth step function reduces the “particle collapse problem” and the “large gradient-jump problem” effectively in the softcore potential (18). Despite that, this study further shows that large dV/dL deviation between antigen-bound and unbound systems is observed at certain λ windows. Excluding such λ windows for the calculation of RBFE tends to reduce the RMSE of TI prediction, suggesting that a comparison of dV/dL distribution may be a method to examine the reliability of TI prediction. However, the causes of large dV/dL deviation and its association with prediction error remain unclear. As shown in **Figure S1C**, mutations with large dV/dL deviation issues tend to enrich small-to-large and charge-changing mutations. We suspect that multiple factors including residual “large gradient-jump problem”, sampling convergence, and mutation-specific structural microenvironment may contribute to the observed large dV/dL deviation. Our TI protocol provides scripts to show the sampling convergence and issues of a TI run by examining the HREMD exchange rate (**Figure S4A**, a higher rate is better) (36), dV/dL distribution (**Figure S4B**), and dV/dL deviation between antigen-bound and unbound systems (**Figure S4C**). In future studies, we will examine whether the optimization of the smooth step function could reduce the prediction error. Ongoing efforts also include a further reduction of the deviation between independent TI repeats by incorporating advanced techniques (e.g., temperature REMD and Arbitrary Degree of Freedom) to HREMD (18). While a single TI run shows an accuracy high enough for mutation screening, averaging over multiple TI runs will be optimal in cases where high accuracy is demanded.

The improved TI method expands the mutational space sampled during *in silico* mutational scanning. Many antibody/antigen interactions involve post-translational modifications (PTMs) (e.g., N-glycosylation, phosphorylation, and tyrosine sulfation). With the improved glycan force field and tools available to easily generate force field parameters for PTMs, the TI method provides a tool to optimize PTM interactions, which cannot be handled by most KB methods. Despite the fact that the performance of TI on other biomolecule systems is still under evaluation, we expect that the current protocol will be fairly accurate for optimizing interactions between diverse biomolecules.

Most KB algorithms [e.g. FoldX (12), mmCSM-AB (10), and SAAMBE-3D (13)] train empirical or statistical models to calculate free energy changes (e.g. electrostatic and van der Waals interactions and solvation) upon mutation. The dataset used for model training are databases including SKEMPI (37), AB-BIND (38), and PROXiMATE (39), which contain binding affinity of approximately 7,000 protein mutations measured by experimental assays. Our results indicate that KB methods have a high accuracy at finding deleterious rather than beneficial mutations, probably because the training databases contain imbalanced numbers of beneficial and detrimental mutations (only approximately 10% beneficial mutations and approximately 50% alanine mutations). While computational approaches have been used to balance the training dataset for mmCSM-AB (10), this study reveals that their accuracy still has room for improvement. One pitfall is that the sample size of the available beneficial mutations unseen by KB methods for evaluating their performance is small. Accumulation of more mutational data, especially beneficial mutations, from *in vivo* and *in vitro* affinity maturation studies will be a critical and limiting factor for evaluating and advancing KB methods. Nonetheless, the high accuracy of the KB methods at identifying deleterious mutations is still very useful in protein design. We demonstrate that the incorporation of both KB and TI methods, by taking advantage of the high efficiency of KB methods and the high accuracy of TI, is effective at finding beneficial mutations. This study provides proof-of-concept validation by combining FoldX, Rosetta, and TI. The incorporation of other KB methods will be an important next step to improve the accuracy of the KB-TI strategy.

The development of highly potent 10-40-like antibodies is an important goal of anti-sarbecovirus therapeutics and vaccine design (28). This study reveals that the substantial binding affinity improvement of antibody 10-40 does not increase the neutralization potency of the omicron variant to a very low level. One hypothesis is that the spike trimer of the omicron variants evolves to have more RBD protomers in the down conformation (35), which buries the 10-40 epitope inside the spike trimer. In such cases, both *in vivo* and *in vitro* affinity maturation may not result in substantial potency improvement. Thus, this study suggests that epitope inaccessibility may be a roadblock to further improving the potency of 10-40-like antibodies against SARS-CoV-2 variants by *in silico* design or vaccination.

## Materials and methods

### Antibody/antigen complex preparation

For each antibody/antigen system in the TI validation dataset (**Table S1**), the antibody Fv domain and the epitope domain of the antigen from the experimentally determined structures were used as the starting complex for RBFE calculations of TI and KB methods. Water molecules beyond 5Å of protein atoms in the original PDB structure were excluded.

The VRC01/RSC3 complex for TI RBFE calculation was modeled using the VRC01/gp120 crystal structure as a template (PDB:3NGB). Modeller v9.16 with default parameters was used to model the complex (40). Positions 76-87 are deleted in RSC3 but were kept in the modeled RSC3 structure to prevent chain break.

Antibody 10-40 Fab/RBD_D614G_ complex downloaded from the protein data bank (PDB) database (PDB IDs: 7SD5) was used for the RBFE calculations of 10-40 mutations against the RBD_D614G_. Because the 10-40/RBD_BA5_ complex structure is unavailable and a conformation change is observed at epitope positions of 370 to 376 in the antibody- free state (35), we modeled the 10-40/RBD_BA5_ complex and relaxed the structure using MD simulation. Briefly, Modeller v9.16 with default parameters was used to model the 10-40/Omicron RBD_BA5_ complex (40). An antibody MD simulation pipeline was used to perform 20ns MD simulation (41). Briefly, the tleap program was used to add a 10 angstrom (Å) cubic water box to the system to neutralize the charge and generate topology and parameter files for MD simulation. Amber20 with the amber14 and GLYCAM_06j-1 force fields (42, 43) was used to perform 20ns isothermal isobaric MD simulation with a 2 fs time step (after 10,000 steps of solution energy minimization, 10,000 steps of whole system energy minimization, 5ns for heating from 0k to 300k, and 2ns of equilibration in the isothermal isovolumetric ensemble) (41). The last snapshot from the MD simulation was used for FoldX, Rosetta, and TI RBFE calculations of 10-40 mutations against RBD_BA5_. Water molecules beyond 5Å of protein atoms were excluded.

### Thermodynamics integration

Amber20 was used to perform TI simulations with the “one-step” transformation using pmemd.cuda.MPI for the HREMD runs and pmemd.cuda for the conventional runs (44). The structures described in the above section were used as the starting complex for the TI simulation. Reduce was used to assign protonation states for titratable residues to the pH of the experimental binding affinity assay and to rotate the side chains of ASN, GLN, and HIS. Tleap (45) was used to build mutations, add disulfide bonds and a 6- angstrom cubic periodic solvent box (TIP3P model, system sizes are listed in **Table S2**), and generate topology files for antibodies and antibody/antigen complexes. Na+ or Cl- was added to neutralize the charge in the system. ParmEd was used to remove redundant bonding terms. For each system, 10,000 steps of solvent minimization (solute is restrained with a force of 25kcal•mol^-1^•Å^-2^) and 10,000 steps of whole system minimization (5kcal•mol^-1^•Å^-2^ restraint on solute) were performed. The solute restraint of 5kcal•mol^-1^•Å^-2^ was also applied to the following steps. The system was heated from 100K to 298K in 50ps. Next, a 100ps simulation in the Isothermal–isobaric (NPT) ensemble was performed to adjust the density of the system, followed by five short simulations (each 200ps, canonical NVT ensemble) to gradually reduce the solute restraint to zero. Next, we equilibrated the system for each λ window (0.00922, 0.04794, 0.11505, 0.20634, 0.31608, 0.43738, 0.56262, 0.68392, 0.79366, 0.88495, 0.95206, and 0.99078) using a strategy described previously (19). Briefly, 1ns equilibration for λ 0.56262 was first performed. The final snapshot was used as the starting configuration for 1ns equilibration of adjacent λ windows (e.g., 0.43738 and 0.68392). Finally, 3ns or 5ns HREMD or independent production run was performed with energy information saved and state exchange attempts between adjacent λ windows every 2ps. Both equilibration and production runs were performed in the NVT ensemble with Langevin dynamics to control temperature and with a 1fs time step. The mutated residues were included in the softcore region with the smoothstep softcore potential for dV/dλ calculation. For predicting ΔΔG of combination mutations, the mutations were introduced sequentially to the antibody with the last snapshot of the TI simulation of the former mutation used as input structure for the TI run of the latter mutation.

We used the trapezoidal rule to analyze the TI gradients (dV/dλ) from the production run with the first 0.5ns excluded as equilibration. The cumulative average of dV/dλ values over time was plotted to check sampling convergence, with TI runs not converged discarded. For each TI run, mean dV/dλ and standard deviation (SD) were calculated for each λ window. A λ window was excluded if its mean dV/dλ of the unbound TI run deviates from the mean dV/dλ of the bound TI run by over one SD, and vice versa. No data decorrelation was performed for ΔΔG calculation.

### Prediction of the effects of mutations by FoldX, Rosetta, SAAMBE-3D, ddg-predictor, and mmCSM-AB

For all antibody/antigen systems, the structures were first energy minimized using the Repair PDB module of FoldXv5, and the optimized structures were then used for FoldX free energy calculation with default settings. The fast relax protocol of Rosetta (2021.07+release.c48be26) was used to minimize the energy of antibody/antigen complexes before the Rosetta free energy calculation using the cartesian_ddg protocol with default settings (33). The energy minimized 10-40 mutants from Rosetta was also used to calculate free energy by ddg-predictor (34). For SAAMBE-3D (13) and mmCSM-AB (10), no energy minimization was performed and the default parameters were used.

### Predicting beneficial mutations of antibody 10-40

The 10-40/RBD_D614G_ structure shows antibody 10-40 contains 28 paratope residues from both heavy and light chains (identified by PISA (46)). The structures of antibody 10-40/RBD complexes (experimental structure of 10-40/RBD_D614G_ and modeled 10-40/RBD_BA5_) were energy minimized using the Repair PDB module and the fast relax protocol of FoldX and Rosetta, respectively. Mutations to all other amino acids at the 28 paratope positions were first ranked by FoldX and Rosetta, and mutations with predicted ΔΔG <0.5kcal/mol by either FoldX or Rosetta against both RBD_D614G_ and RBD_BA5_ and FoldX stability change <1.0 kcal/mol were kept. TI was applied to exclude deleterious mutations against RBD_D614G_ and RBD_BA5_. TI- identified beneficial mutations that were then ranked by the RBFE. Cysteine mutations were excluded due to their high reactivity with free cysteines in a cell culture medium (47). Mutations resulting in the N-glycosylation site (NXS/T motif, X cannot be Pro) were also excluded.

### Molecular dynamics simulation and trajectory analysis

Antibody 10-40 mutations were introduced to the 10-40Fab/RBD_D614G_ complex (PDB IDs: 7SD5) and the modeled 10-40Fab/RBD_BA5_ complex (see above) for MD simulations to study their effects on antibody and antigen interactions. A published antibody MD simulation pipeline was used to perform MD simulation (41). Briefly, the tleap program was used to introduce mutations to the antibody, add a 10 angstrom (Å) cubic water box to the system, neutralize the charge, and generate topology and parameter files for MD simulation. Amber20 with the amber14 and GLYCAM_06j-1 force fields (42, 43) was used to perform 200ns isothermal isobaric MD simulation per run (after 10,000 steps of solution energy minimization, 10,000 steps of whole system energy minimization, 5ns for heating from 0k to 300k, and 10ns of equilibration in the isothermal isovolumetric ensemble) on each Fab variant (41).

A master analysis script (Traj.R) was used to perform MD trajectory analysis (41). Briefly, for each MD production run, snapshots of the first 100ns were discarded as equilibration. Each snapshot was superimposed to the first snapshot using Cα atoms of the heavy and light chain variable domains and root-mean-square deviation (RMSD) was calculated to determine simulation convergence (48). For each snapshot, we quantified the sampled distributions of the torsion and tilting angles and distance between V_H_ and V_L_ using ABangle recompiled in R (49), elbow angle by PyMOL, buried ASA, and hydrogen bond networks between domain interfaces using PISA (46). All statistical analyses were performed in R.

### Cloning of antibodies and SARS-CoV-2 RBD variants

Genes encoding for the heavy and light chains of antibody 10-40 were inserted separately into pcDNA3.4 plasmids. Respective genes for the SARS-CoV-2 RBD variants (wild type and Omicron BA5) were cloned into mammalian expression vector pLXM followed by a C-terminal octa-histidine tag. All the cloning was done using T4 ligase (NEB).

### Site-directed Mutagenesis using double-primer PCR

Site-directed mutagenesis was performed as previously described (41). The 10-40 mutants were generated using Pfu Ultra II polymerase in a protocol that employed both forward and reverse primers in the same PCR reaction for 18 cycles. The PCR products were denatured and then reannealed. The non-mutated methylated parental plasmid was digested with DpnI (NEB), and the remaining plasmids were transformed into E. coli cells. For each transformation, five colonies were selected at random and grown overnight in 5 ml LB + Ampicillin medium at 37°C. The plasmids were isolated using the Spin miniprep kit (Qiagen, Germany) and sequenced to obtain the desired mutants. All the pLXM plasmids encoding SARS-CoV-2 RBD variants were transformed in a similar manner.

### Expression and purification of antibody 10-40 mutants and SARS-CoV-2 RBD variants

Recombinant 10-40 antibodies were transiently expressed in Expi293F cells (Thermo Fisher Scientific) in a chemically defined, serum-free medium using the ExpiFectamine™ 293 transfection Kit according to manufacturer’s instructions by the cotransfection of the heavy chain (VH+CH1+CH2+CH3) and light chain (VL+CL)- expressing plasmids. Similarly, SARS-CoV-2 RBD variants (wild type and Omicron BA5) were transfected separately in Expi293F cells using expifectamine. The cell cultures were incubated in a 37°C shaker at 125 rpm under 8% CO2. Supernatants were collected five days after transfection. Antibodies were purified by affinity chromatography using rProtein A Sepharose (Cytiva). The RBD variants were purified by using Ni-NTA IMAC Sepharose 6 Fast Flow resin (GE Healthcare) nickel affinity chromatography. All proteins were further purified by size exclusion chromatography (SEC) using a Superdex 200 increase column in 10 mM Tris pH 8.0, 150 mM NaCl for all 10-40 antibody mutants and 10 mM HEPES, pH7.4, 150 mM NaCl for the RBD variants.

### Surface plasmon resonance

SPR binding assays were performed using a Biacore T200 biosensor equipped with a Series S CM5 chip (Cytiva) at 25°C in an HBS-EP+ buffer (10 mM HEPES, 150 mM NaCl, 3 mM EDTA, 0.05% P-20, pH 7.4). IgGs were captured to the chip surface using immobilized protein A (Cytiva) over all four flow cells. Each IgG antibody was captured over independent flow cells at 2μg/mL at a capture level of approximately 200 RU. A surface without captured IgG served as a reference control. RBD antigens were prepared in a running buffer using a three-fold dilution series at five concentrations ranging from 0.62 to 50nM, using a 150s association time and 600s dissociation time at 30μL/min. At the end of each cycle, the anti-IgG surface was regenerated using 10 mM glycine pH 1.5. Blank buffer cycles were performed by injecting running buffer instead of RBD to remove systematic noise from the binding signal. The resulting data were processed and fit to a 1:1 binding model using Biacore Evaluation Software. Each concentration series were tested in triplicate.

### Pseudovirus production

Pseudoviruses were produced in the vesicular stomatitis virus (VSV) background, in which the native glycoprotein was replaced by that of SARS-CoV-2 and its variants, as previously described (50). In brief, HEK293T cells were transfected with a spike expression construct with 1 mg mL-1 polyethylenimine (PEI) and cultured overnight at 37 °C under 5% CO2 and then infected with VSV-G pseudotyped ΔG-luciferase (G*ΔG-luciferase, Kerafast) one day post-transfection. After 2 h of infection, cells were washed three times, changed to fresh medium, and then cultured for approximately another 24 h before the supernatants were collected, clarified by centrifugation, and aliquoted and stored at -80°C for further use.

### Pseudovirus neutralization assay

All viruses were first titrated to normalize the viral input between assays. Heat-inactivated sera or antibodies were first serially diluted in a medium in 96-well plates in triplicate, starting at 1:100 dilution for sera and 10 µg mL^−1^ for antibodies. Pseudoviruses were then added and the virus–sample mixture was incubated at 37°C for 1 h. Vero-E6 cells were then added at a density of 3 × 10^4^ cells per well and the plates were incubated at 37°C for approximately 10 h. Luciferase activity was quantified using the Luciferase Assay System (Promega) according to the manufacturer’s instructions using SoftMax Pro v.7.0.2 (Molecular Devices). Neutralization curves and IC_50_ values were derived by fitting a nonlinear five-parameter dose-response curve to the data in GraphPad Prism v.9.2.

## Data availability statement

The original contributions presented in the study are included in the article/**Supplementary Material**. Further inquiries can be directed to the corresponding author.

## Author contributions

ZS designed the research. ZS developed the TI pipeline and analyzed the data. JB produced the antibodies and antigens. ZS and YG performed antibody 10-40 saturation mutagenesis. ZS performed the SPR measurement. DH, MW, and ZL performed pseudovirus neutralization. ZS wrote the paper, and all authors reviewed, commented on, and approved the manuscript. All authors contributed to the article and approved the submitted version.
