# Supplementary material for: An optimized thermodynamics integration protocol for identifying beneficial mutations in antibody design
Source: Front Immunol. 2023 May 19;14:1190416. doi: 10.3389/fimmu.2023.1190416 (PMC10235760; doi:10.3389/fimmu.2023.1190416)
Supplement: Supplementary file 1 [file Presentation_1.pptx]

## Slide 1
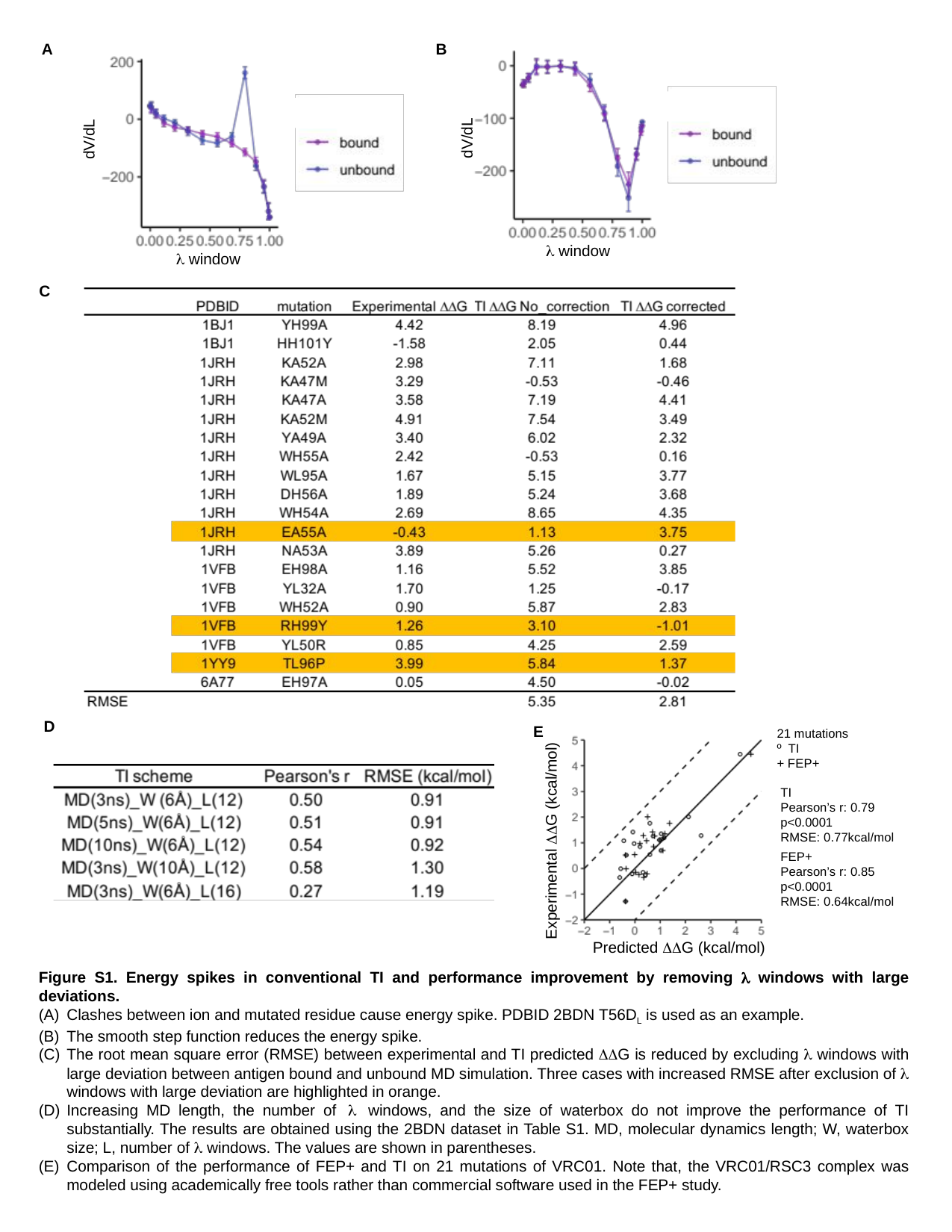

B
A
dV/dL
dV/dL
l window
l window
C
D
E
21 mutations
º TI
+ FEP+
TI
Pearson’s r: 0.79
p<0.0001
RMSE: 0.77kcal/mol
Experimental DDG (kcal/mol)
FEP+
Pearson’s r: 0.85
p<0.0001
RMSE: 0.64kcal/mol
Predicted DDG (kcal/mol)
Figure S1. Energy spikes in conventional TI and performance improvement by removing l windows with large deviations.
Clashes between ion and mutated residue cause energy spike. PDBID 2BDN T56DL is used as an example.
The smooth step function reduces the energy spike.
The root mean square error (RMSE) between experimental and TI predicted DDG is reduced by excluding l windows with large deviation between antigen bound and unbound MD simulation. Three cases with increased RMSE after exclusion of l windows with large deviation are highlighted in orange.
Increasing MD length, the number of l windows, and the size of waterbox do not improve the performance of TI substantially. The results are obtained using the 2BDN dataset in Table S1. MD, molecular dynamics length; W, waterbox size; L, number of l windows. The values are shown in parentheses.
Comparison of the performance of FEP+ and TI on 21 mutations of VRC01. Note that, the VRC01/RSC3 complex was modeled using academically free tools rather than commercial software used in the FEP+ study.

## Slide 2
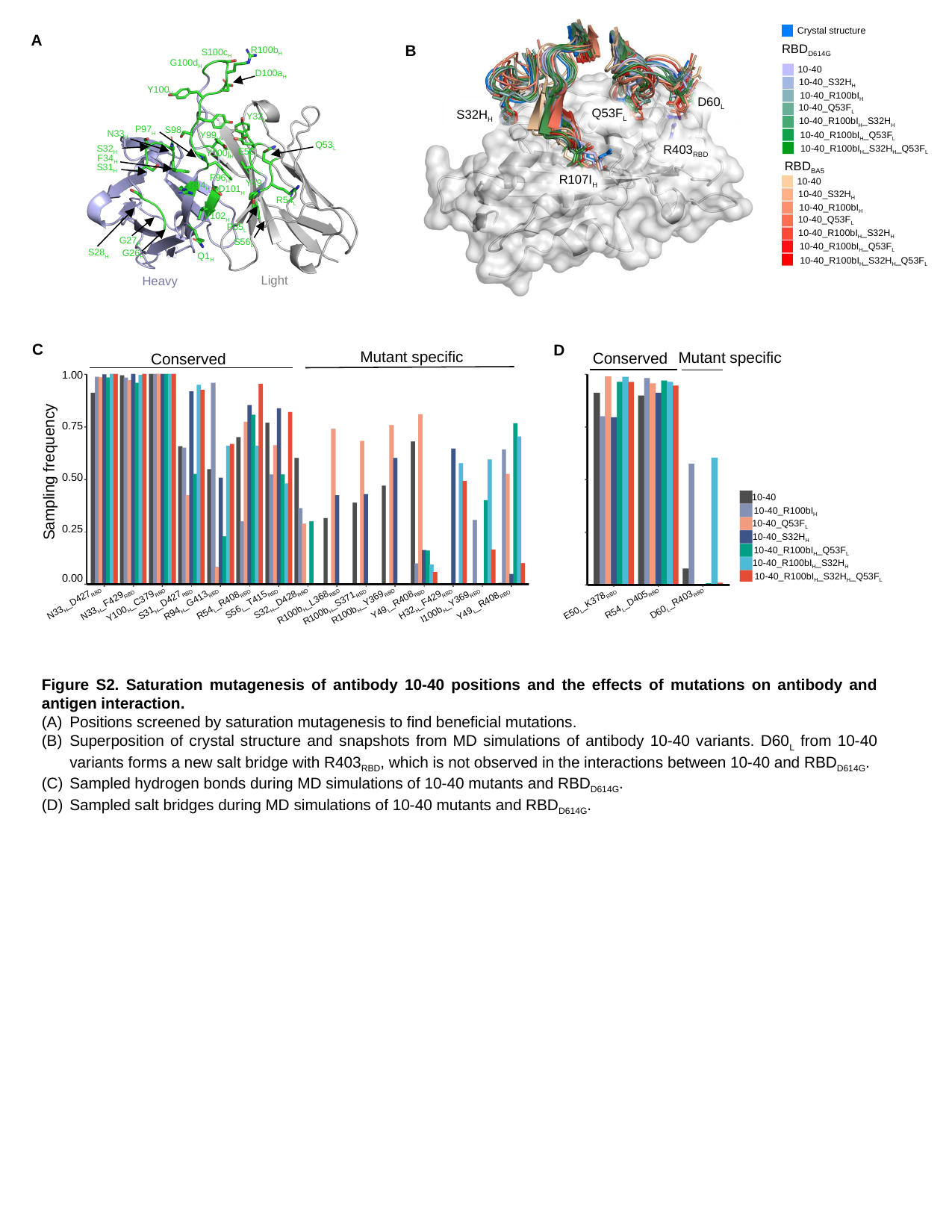

Crystal structure
A
B
RBDD614G
R100bH
S100cH
G100dH
10-40
10-40_S32HH
10-40_R100bIH
10-40_Q53FL
10-40_R100bIH_S32HH
10-40_R100bIH_Q53FL
10-40_R100bIH_S32HH_Q53FL
D100aH
Y100H
D60L
Q53FL
S32HH
Y32L
P97H
S98H
N33H
Y99H
R403RBD
Q53L
S32H
E50L
Y100jH
RBDBA5
F34H
S31H
R107IH
10-40
10-40_S32HH
10-40_R100bIH
10-40_Q53FL
10-40_R100bIH_S32HH
10-40_R100bIH_Q53FL
10-40_R100bIH_S32HH_Q53FL
F96H
Y49L
R94H
D101H
R54L
V102H
P55L
G27H
S56L
S28H
G26H
Q1H
Light
Heavy
C
D
Mutant specific
Mutant specific
Conserved
Conserved
1.00
0.75
Sampling frequency
0.50
10-40
10-40_R100bIH
10-40_Q53FL
0.25
10-40_S32HH
10-40_R100bIH_Q53FL
B
10-40_R100bIH_S32HH
10-40_R100bIH_S32HH_Q53FL
0.00
R54L_D405RBD
D60L_R403RBD
N33H_D427RBD
S32H_D428RBD
S56L_T415RBD
Y49L_R408RBD
S31H_D427RBD
H32H_F429RBD
R54L_R408RBD
R94H_G413RBD
E50L_K378RBD
N33H_F429RBD
Y100H_C379RBD
Y49L_R408RBD
I100bH_Y369RBD
R100bH_L368RBD
R100bH_Y369RBD
R100bH_S371RBD
Figure S2. Saturation mutagenesis of antibody 10-40 positions and the effects of mutations on antibody and antigen interaction.
Positions screened by saturation mutagenesis to find beneficial mutations.
Superposition of crystal structure and snapshots from MD simulations of antibody 10-40 variants. D60L from 10-40 variants forms a new salt bridge with R403RBD, which is not observed in the interactions between 10-40 and RBDD614G.
Sampled hydrogen bonds during MD simulations of 10-40 mutants and RBDD614G.
Sampled salt bridges during MD simulations of 10-40 mutants and RBDD614G.

## Slide 3
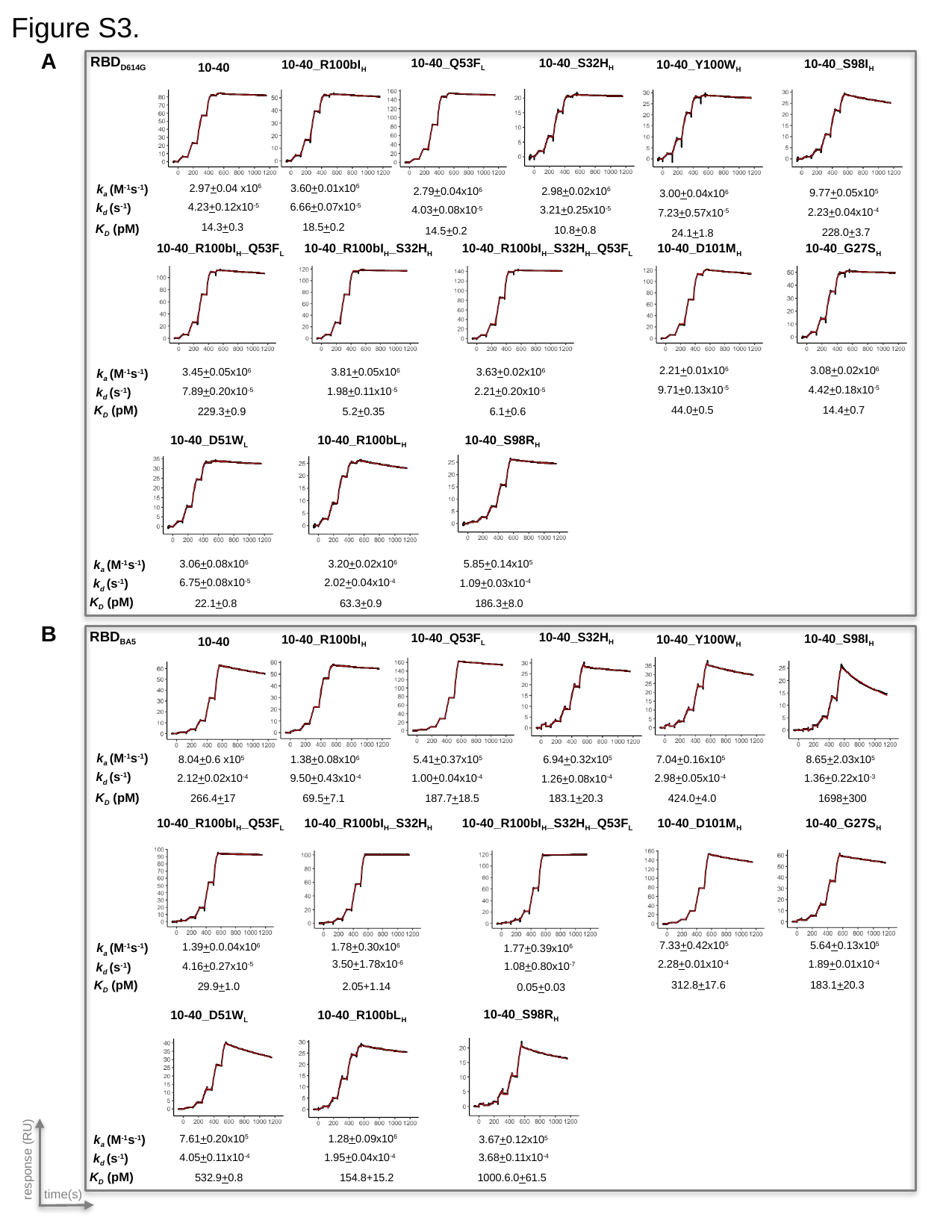

# Figure S3.
A
RBDD614G
10-40_S32HH
10-40_Q53FL
10-40_S98IH
10-40_Y100WH
10-40_R100bIH
10-40
3.60+0.01x10^6^
6.66+0.07x10^-5^
18.5+0.2
ka (M-1s-1)
2.97+0.04 x10^6^
4.23+0.12x10^-5^
14.3+0.3
2.98+0.02x10^6^
3.21+0.25x10^-5^
10.8+0.8
2.79+0.04x10^6^
4.03+0.08x10^-5^
14.5+0.2
9.77+0.05x10^5^
2.23+0.04x10^-4^
228.0+3.7
3.00+0.04x10^6^
7.23+0.57x10^-5^
24.1+1.8
kd (s-1)
KD (pM)
10-40_R100bIH_Q53FL
10-40_R100bIH_S32HH
10-40_R100bIH_S32HH_Q53FL
10-40_D101MH
10-40_G27SH
2.21+0.01x10^6^
9.71+0.13x10^-5^
44.0+0.5
3.08+0.02x10^6^
4.42+0.18x10^-5^
14.4+0.7
ka (M-1s-1)
3.45+0.05x10^6^
7.89+0.20x10^-5^
229.3+0.9
3.81+0.05x10^6^
1.98+0.11x10^-5^
5.2+0.35
3.63+0.02x10^6^
2.21+0.20x10^-5^
6.1+0.6
kd (s-1)
KD (pM)
10-40_D51WL
10-40_S98RH
10-40_R100bLH
ka (M-1s-1)
3.06+0.08x10^6^
6.75+0.08x10^-5^
22.1+0.8
3.20+0.02x10^6^
2.02+0.04x10^-4^
63.3+0.9
5.85+0.14x10^5^
1.09+0.03x10^-4^
186.3+8.0
kd (s-1)
KD (pM)
B
RBDBA5
10-40_S32HH
10-40_Q53FL
10-40_S98IH
10-40_Y100WH
10-40_R100bIH
10-40
ka (M-1s-1)
8.04+0.6 x10^5^
2.12+0.02x10^-4^
266.4+17
1.38+0.08x10^6^
9.50+0.43x10^-4^
69.5+7.1
5.41+0.37x10^5^
1.00+0.04x10^-4^
187.7+18.5
7.04+0.16x10^5^
2.98+0.05x10^-4^
424.0+4.0
8.65+2.03x10^5^
1.36+0.22x10^-3^
1698+300
6.94+0.32x10^5^
1.26+0.08x10^-4^
183.1+20.3
kd (s-1)
KD (pM)
10-40_R100bIH_Q53FL
10-40_R100bIH_S32HH
10-40_R100bIH_S32HH_Q53FL
10-40_D101MH
10-40_G27SH
7.33+0.42x10^5^
2.28+0.01x10^-4^
312.8+17.6
5.64+0.13x10^5^
1.89+0.01x10^-4^
183.1+20.3
ka (M-1s-1)
1.39+0.0.04x106
4.16+0.27x10^-5^
29.9+1.0
1.78+0.30x10^6^
3.50+1.78x10^-6^
2.05+1.14
1.77+0.39x10^6^
1.08+0.80x10^-7^
0.05+0.03
kd (s-1)
KD (pM)
10-40_S98RH
10-40_D51WL
10-40_R100bLH
ka (M-1s-1)
7.61+0.20x10^5^
4.05+0.11x10^-4^
532.9+0.8
1.28+0.09x10^6^
1.95+0.04x10^-4^
154.8+15.2
3.67+0.12x10^5^
3.68+0.11x10^-4^
1000.6.0+61.5
kd (s-1)
response (RU)
KD (pM)
time(s)

## Slide 4
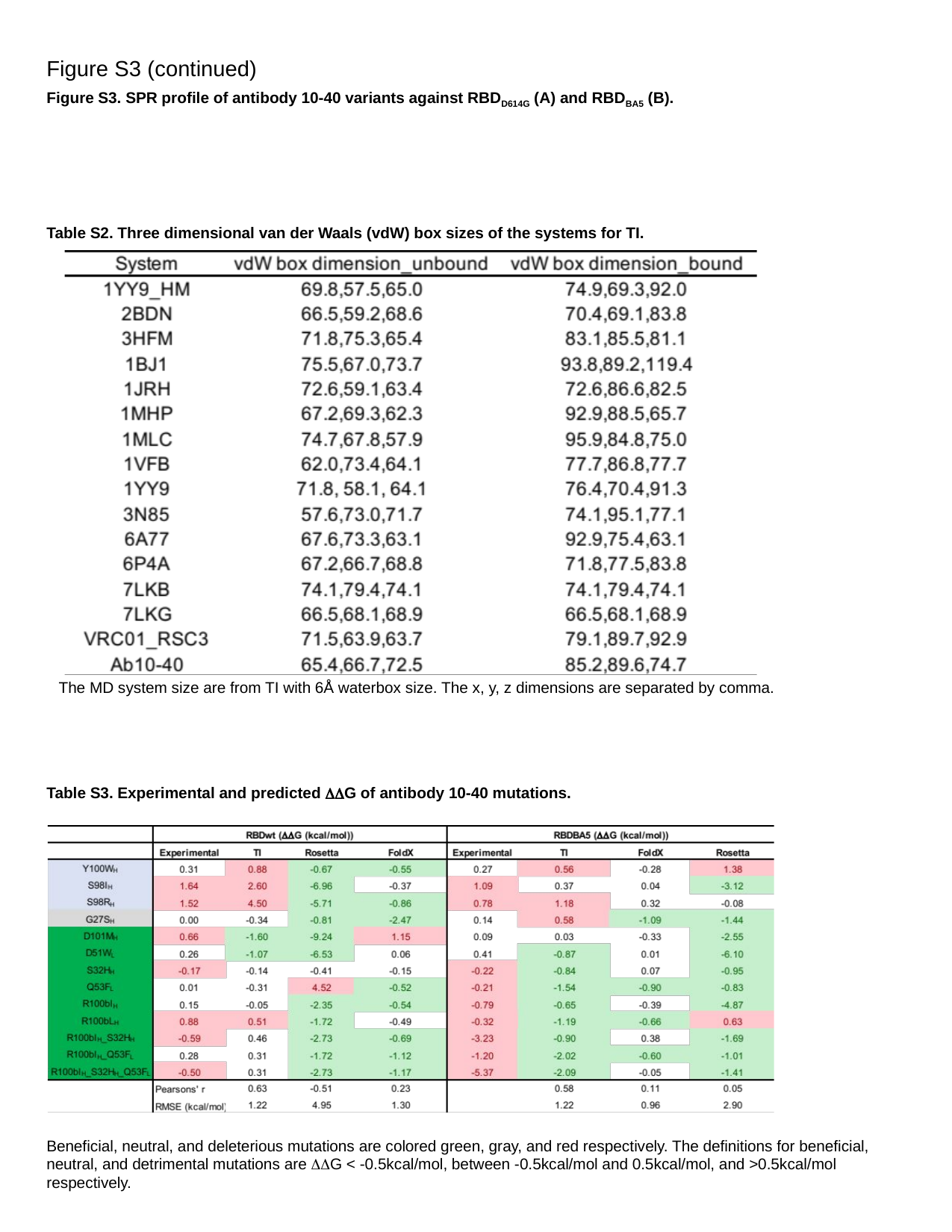

# Figure S3 (continued)
Figure S3. SPR profile of antibody 10-40 variants against RBDD614G (A) and RBDBA5 (B).
Table S2. Three dimensional van der Waals (vdW) box sizes of the systems for TI.
The MD system size are from TI with 6Å waterbox size. The x, y, z dimensions are separated by comma.
Table S3. Experimental and predicted DDG of antibody 10-40 mutations.
Beneficial, neutral, and deleterious mutations are colored green, gray, and red respectively. The definitions for beneficial, neutral, and detrimental mutations are DDG < -0.5kcal/mol, between -0.5kcal/mol and 0.5kcal/mol, and >0.5kcal/mol respectively.

## Slide 5
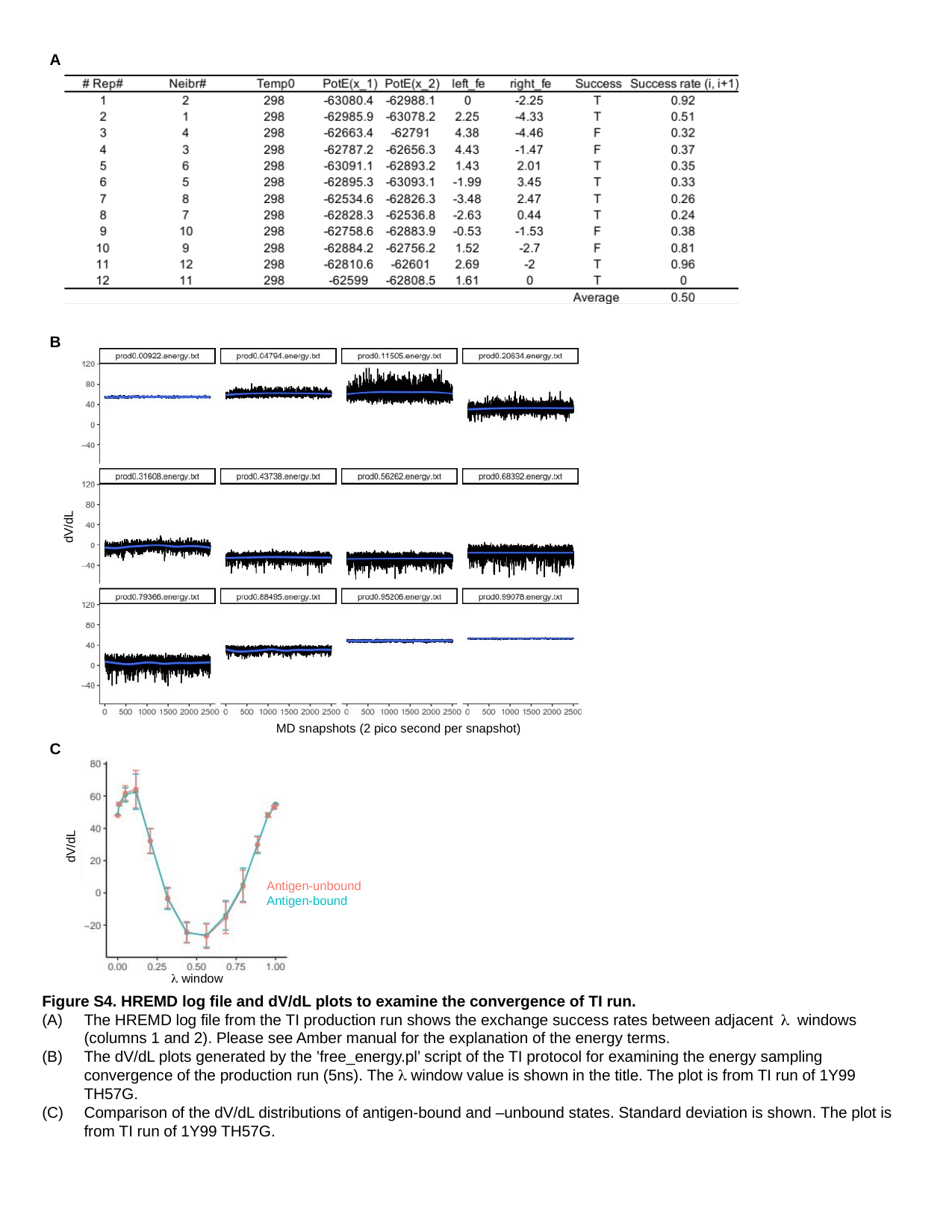

A
B
dV/dL
MD snapshots (2 pico second per snapshot)
C
dV/dL
Antigen-unbound
Antigen-bound
l window
Figure S4. HREMD log file and dV/dL plots to examine the convergence of TI run.
The HREMD log file from the TI production run shows the exchange success rates between adjacent l windows (columns 1 and 2). Please see Amber manual for the explanation of the energy terms.
The dV/dL plots generated by the ’free_energy.pl’ script of the TI protocol for examining the energy sampling convergence of the production run (5ns). The l window value is shown in the title. The plot is from TI run of 1Y99 TH57G.
Comparison of the dV/dL distributions of antigen-bound and –unbound states. Standard deviation is shown. The plot is from TI run of 1Y99 TH57G.
